# Parental Burnout and Its Antecedents among Same-Sex and Different-Sex Families

**DOI:** 10.3390/ijerph19137601

**Published:** 2022-06-21

**Authors:** Jorge Gato, Anne Marie Fontaine, Filipa César, Daniela Leal, Isabelle Roskam, Moïra Mikolajczak

**Affiliations:** 1 Faculty of Psychology and Education Sciences, University of Porto, 4200-135 Porto, Portugal; fontaine@fpce.up.pt (A.M.F.); filipa.cesar@gmail.com (F.C.); danielaleal.psic@gmail.com (D.L.); 2 Department of Psychology, UCLouvain, 1348 Ottignies-Louvain-la-Neuve, Belgium; isabelle.roskam@uclouvain.be (I.R.); moira.mikolajczak@uclouvain.be (M.M.)

**Keywords:** parental burnout, same-sex families, gender

## Abstract

Parental burnout (PB) results from a chronic imbalance between risks and resources and has severe and extended consequences on the wellbeing of parents and their children. Because same-sex (SS) and different-sex (DS) families face partially different stressors (e.g., SS parents are more stigmatized) but have also partially different resources (e.g., more egalitarian task sharing in SS couples), the current research aimed to investigate whether PB differs or not according to family type. Two studies were conducted. In study 1, family type differences in PB were explored among 114 demographically matched SS and DS families from 18 countries. Study 2 further explored the predictive value of family type, age, gender, and balance between risks and resources (BR2) in PB, using a sample of 222 matched SS and DS families. Parental burnout was not associated with family type in either study. Although differentially composed, the global BR2 score did not differ across family type and was a significant predictor of all PB dimensions, while controlling for the effect of family type, age, and gender. Thus, in accordance with reviewed studies, parental sexual identity was not associated with family functioning. Future studies should investigate the impact of specific risks and resources (e.g., social support from chosen social networks or legal climate) on PB levels among SS families.

## 1. Introduction

The burnout syndrome, a concept initially investigated in the workplace realm [1], was recently studied in the family sphere [2,3,4,5]. Parental burnout includes experiences of exhaustion related to one’s parental role, emotional distancing from one’s children, feelings of being fed up with one’s parental role, and contrast with how the parent used and wanted to be [6]. It is determined by a multitude of factors, such as socio-demographic characteristics, particularities of the child, parents’ stable traits, parenting factors, and family functioning [7] and has severe consequences on the mental and physical health of parents and on their children [3,7,8,9,10]. This syndrome has been mostly examined in families headed by different-sex couples. However, the number of same-sex parent families is on the rise, calling out for increasing attention in the wider field of family science [11] and in the specific realm of parental burnout.

### 1.1. Same-Sex Families: Thriving in Adversity

Sexual-minority parents experience more social stigma and less social support from families of origin than their heterosexual counterparts [12,13,14,15]. In the same vein, legal vulnerability also constitutes an increased risk for same-sex families [16]. However, legal and social support seem to buffer against the negative impact of stigma [17,18]. For instance, Lick and colleagues [19] found better psychological outcomes for children raised in same-sex families in areas of the USA with antidiscrimination laws. Still, decades of research with same-sex families have pointed out to more similitudes than differences between same-sex and different-sex families both regarding parenthood [20] and child development and well-being [20,21,22,23,24,25]. Indeed, when differences are found across groups of same-sex versus different-sex parent families, they are accounted for by variables other than sexual-minority status, including lower SES and family transitions [26]. Deficit models of same-sex parenthood have also been recently challenged. In this regard, the population-level study of Kabatek and Perales (2021) has reported that children in same-sex-parented families outperform children in different-sex-parented families on multiple indicators of academic performance [27]. Moving beyond a comparative paradigm [28,29], some studies investigating processes within same-sex parent families have highlighted specific resources. For instance, these parents seem to carefully choose their social networks [30], to have positive attitudes towards diversity [31,32], and to engage with children in unique socialization practices that may equip them with specific skills to cope with stigma [32,33]. All these protective mechanisms seem to account for the well-being of these parents and their children and to compensate for the existing social and legal risks.

### 1.2. Parental Burnout: An Overview

Parental burnout is attracting more and more attention from both scientists and media [3]. This is not surprising as recent research showed that parental burnout is a prevalent condition (affecting 5 to 9% of Western parents; [5]) that has severe and extended consequences. These include increased suicidal ideation among parents [2] and also dysregulated hypothalamic–pituitary–adrenal axis (HPA) [3,8], which may help account for the somatic complaints and sleep disorders reported by burned-out parents [10]. In addition to affecting the parents, parental burnout increases the risk of child neglect and/or violence against one’s children [2]. Psychological interventions targeted at parental burnout decrease violence and neglect in a manner proportional to the decrease in parental burnout and the HPA axis activity normalizes [8,34] (for an up-to-date review of the parental burnout syndrome, its antecedents, consequences, and treatment please see [3] and [35]).

Parental burnout is the result of an imbalance between demands (risk factors) and resources (protection factors) [36]. Demands/risks are those factors which significantly increase parental stress, such as parental perfectionism [37], low emotional intelligence [7], poor child-rearing practices [7], countless parental duties and chores, lack of support from the co-parent [7,38], lack of family support (family support, nurseries, etc.) [35], among others. Conversely, resources/protection can be defined as factors that help to significantly alleviate parental stress, including parental self-compassion, high emotional intelligence, good childrearing practices, time for leisure, positive co-parenting, external support, etc. Thus, resources are not the absence of risks, but the opposite of risks. According to this theory, parental burnout threatens any parent who accumulates too many risks without enough compensatory resources [3,36]. Furthermore, research showed that psychological variables (e.g., personality, parenting practices, and family functioning) explain a greater proportion of parental burnout than sociodemographic characteristics (e.g., gender, children’s age) [5,7,39]. 

### 1.3. The Current Research

Our goal in this work was to explore parental burnout and its antecedents (more specifically, balance between risks and resources) among same-sex and different-sex families. Because same-sex and different-sex families face partially different stressors (e.g., same-sex families are still stigmatized) but have also partially different resources (e.g., more egalitarian task sharing in same-sex families) and because parental sexual identity is not associated with parenting dynamics nor with children’s wellbeing, we expected a priori no differences in parental burnout between different-sex and same-sex families (Hypothesis 1). Just as we anticipate no differences in parental burnout as a function of family type, the same applies to balance between risks and resources (Hypothesis 2). Finally, we aim to test the predictive value of family type, gender, and balance between risks and resources on parental burnout. We expect parental burnout to be predicted by balance between risks and resources, but not by family type and gender (Hypothesis 3). Two studies were conducted to test these hypotheses. The small number of participants in Study 1 imposed some caution on the generalizability of results. Resorting to a larger sample, Study 2 allowed for a more stringent examination of the hypotheses. Study 1 used a sample of 114 matched same-sex and different-sex families from 18 countries and Study 2 used a sample of 220 matched same-sex and different-sex families (mostly from Belgium).

## 2. Materials and Methods 

### 2.1. Study 1: Preliminary Investigation 

#### 2.1.1. Participants 

The data of this study were draw from a larger sample of 16,889 parents collected by the International Investigation of Parental Burnout (https://www.burnoutparental.com/international-consortium, accessed on 25 March 2022) between 2017 and 2019. Of these participants, 59 were part of a same-sex household and 57 were extracted for the present study. Two participants were excluded based on inconsistent/incomplete responses (one reported 20 children living at home, and another did not provide data about children’s age). Given our research goal, these 57 participants belonging to a same-sex household were strictly matched based on their sociodemographic characteristics (country, gender, age, education level, number of children at home, neighbourhood, and paid professional activity), with an equal number of participants randomly selected from a subsample of different-sex families (*n* = 13,207). The participants’ ages ranged from 19 to 58 years, and they were evenly matched regarding gender and country. The final sample on which the analyses of the current study were carried thus comprised 114 individuals of which 90 (78.9%) were mothers and 24 (21.1%) were fathers. The majority of the sample (75.4%) came from Europe (Belgium, *n* = 26, Finland, *n* = 18; France, *n* = 18; Sweden, *n* = 12; Germany, *n* = 4; Switzerland, *n* = 4; Austria, *n* = *2*; The Netherlands, *n* = *2*) and the remaining (24.6%) from non-European countries (Chile, *n* = 4; China, *n* = 4; Peru, *n* = 4; Vietnam, *n* = 4; Canada, *n* = 2; Costa Rica, *n* = *2*; Japan, *n* = *2*; Rwanda, *n* = 2; Thailand, *n* = 2; USA, *n* = 2). Sociodemographic characteristics of the total sample and subsamples can be consulted in Table 1. 

#### 2.1.2. Procedure

This study is part of the International Investigation of Parental Burnout (IIPB) project. The survey was translated locally by participating research teams. Participants received an informed consent form prior to starting the questionnaire assuring them that their data would remain anonymous and clarifying that they could withdraw at any time. Parents were eligible to participate in the study if they had (at least) one child still living at home. The main data collection procedure was the online form, but paper-and-pencil were also used to reach respondents without internet access. The online survey dissemination varied from country to country, but mainly focused on institutional communication channels of the various consortium’s teams, media (including social networks), through schools and other educational and/or local institutions (for details about the methodology, see [36]). 

#### 2.1.3. Measures

Sociodemographics: Participants were asked about their gender, country of residence, age, education level, family configuration, number of children at home, type of neighbourhood, and professional status (response options can be consulted in Table 1).

Parental Burnout: Parental burnout was assessed through the Parental Burnout Assessment ([5,6] for measurement invariance across countries, languages, and genders). This 23-item instrument is composed of four subscales: (i) Exhaustion in the parenting role (EX; e.g., “I feel completely run down by my role as a parent”); (ii) Contrast with previous parental self (CO; e.g., “I tell myself that I’m no longer the parent I used to be”); (iii) Feelings of Being Fed Up and loss of pleasure in the parental role (FU; e.g., “I don’t enjoy being with my children anymore”); and (iv) Emotional Distancing from one’s children (ED; e.g., “Outside the usual routines (lifts in the car, bedtime, meals), I’m no longer able to make an effort for my child(ren)”). Items are rated on a 7-point Likert scale: “never” (0), “a few times a year of less” (1), “once a month or less” (2), “a few times a month” (3), “once a week (4), “a few times a week” (5), “every day” (6). Items were summed, such that higher scores reflect greater parental burnout. The internal consistency of the four subscales was acceptable in the overall group and family type subgroups (Cronbach’s alphas between 0.71 and 0.95). 

#### 2.1.4. Statistical Analyses 

In order to reduce selection bias, improve internal validity, and control for the effect of confounding variables, we used the SPSS Case-control Matching tool for matching samples in both studies. This technique allowed us to control the impact of sociodemographic variables on results in order to truly assess the significant impact of parental sexual identity on the analysed outcomes. The equivalence of same-sex and different-sex families groups was confirmed using t and chi-square tests. The normality of the distribution of the continuous variables used in the study was inspected considering the cut-off values of sk < │3│and ku < │7│. Differences in the parental burnout scales were investigated using MANOVA with the continuous score of parental burnout as dependent variable. A power analysis using the G* Power 3.1.9.4 software indicated that a minimum total sample size of 80 people would be needed to detect a medium effect size f2 (V) = 0.25 with a conventional power of 0.95 at 0.05 significance level, using MANOVA with two groups and four response variables. A minimum sample size of 130 participants would be needed to detect a small effect size of 0.15. 

### 2.2. Study 2: Replication and Extension

#### 2.2.1. Participants 

The sample used in Study 2 was more homogeneous in terms of geographical provenience (mostly Belgian, French, and Swiss families) and it included more participants and an assessment of balance between risks and resources. Data were collected from a sample of 3535 parents having at least one child still living at home. Among these 111 same-sex families were matched regarding gender and country with 111 different-sex families selected in the subsample of 1791 biparental families (single-parent families and stepfamilies were not considered). The final sample comprised 222 French-speaking parents of which 192 (86.5%) were women and 30 (13.5%) were men. The participants’ ages ranged from 22 to 66 years. The majority of the participants came from Belgium (*n* = 142; 64%), followed by France (*n* = 42; 18.9%), Switzerland (*n* = 16; 7.2%), Québec (*n* = 20; 9%), and other French speaking countries (*n* = 2; 0.9%). Sociodemographic characteristics of the total sample and subsamples can be consulted in Table 2.

#### 2.2.2. Procedure

Participants were informed about the survey through social networks and with the help of the largest mutual health benefit society in Belgium. In order to avoid (self-)selection bias, participants were not informed that the study was about parental burnout. The study was presented as a study about parental fulfilment and exhaustion. Parents were eligible to participate in the study if they had (at least) one child still living at home. Participants were invited to complete the survey after giving informed consent. The questionnaire was completed anonymously online with the forced option on, ensuring a data set with no missing data. Participants who completed the questionnaire had the opportunity to enter a lottery with a chance of winning €200. Participants who wished to participate in the lottery had to provide their email address, but the latter was automatically disconnected from their questionnaire.

#### 2.2.3. Measures 

Sociodemographics: Participants were asked about the same sociodemographic characteristics as in Study 1. 

Parental Burnout: Parental burnout was measured as described in Study 1. The internal consistency of the four subscales was acceptable in the overall group and sexual identity subgroups (Cronbach’s alphas between 0.66 and 0.95).

Balance between Risks and Resources: This variable was assessed only in this Study by means of the BR2, an instrument created to operationalize the Balance between Parental Risks and Resources Theory [36]. The instrument encompasses 39 bipolar items encompassing 11 levels, i.e., from −5 to +5 including 0. The negative pole represents the risk while the positive pole represents the corresponding resource. For example, “I find it difficult to reconcile my family life and my professional life” (−5); “I can easily reconcile my family life and my professional life”. The global score is computed by summing the 39 items so that positive scores indicate that the parent has more (or heavier) resources than risks, negative scores indicate that the parent has more (or heavier) risks than resources, and zero scores indicate that the parent has the same level of risks and resources. The internal consistency for the scale (Cronbach’s alpha) was 0.94 for the global sample and 0.94 and 0.95 for same-sex families and different-sex households, respectively. 

#### 2.2.4. Statistical Analyses 

Statistical Analyses were carried out as in Study 1. Furthermore, hierarchical regression models on each parental burnout subscale were run, with control variables in Step 1 and balance of risks and resources in Step 2. All scores were kept continuous. A power analysis using the G* Power 3.1.9.4 software indicated that a minimum total sample size of 80 participants would be needed to detect a medium effect size = 0.25 with a conventional power of 0.95 at 0.05 significance level, using Linear Multiple Regression with four predictors. A minimum sample size of 129 participants would be needed to detect a small effect size of 0.15. Because the study includes 222 matched families (111 same-sex and 111 different-sex), power is sufficient to compare the two groups. We used Tolerance and VIF as multicollinearity indexes; the most common cut-off employed is a tolerance value > 0.10 corresponding to a VIF < 10. 

## 3. Results

### 3.1. Study 1 

Parental burnout subscales were distributed within the normality range regarding both skewness (1.23 to 1.91) and kurtosis (0.75 to 3.75). No differences were found in sociodemographic characteristics (age, education level, number of children at home, neighbourhood, and paid professional activity) as a function of family type (Table 1). In accordance with H1, the main effect of family type was not significant on all subscales of parental burnout, Pillai’s trace = 0.04, F (4, 109) = 1.00, *p* = 0.414, ηp2 = 0.04, observed power = 0.31. These results need to be interpreted with caution given the small sample and corollary low power of the analysis. 

### 3.2. Study 2

Parental burnout subscales were distributed within the normality range (2.12 < sk < 2.54; 0.90 < ku < 5.68); the value of kurtosis for the subscale contrast was slightly above the recommend value (7.45), but we still decided to include it in the analyses. The BR^2^ scale was normally distributed (sk = −0.26 and ku = 0.71). No differences were found in sociodemographic characteristics (education level, number of children at home, income, and work status) as a function of family type, except for age, with same-sex parents being older than their different-sex peers (Table 2). 

**Table 2:** Sociodemographic Variables as a Function of Family Type (Study 2).

	*M (SD)*			
	Total (*n* = 222)	Same-Sex Families *(n =* 111*)*	Different-Sex Families *(n =* 111*)*	*p*
Age	40.1 (8.3)	42.0 (8.7)	38.2 (7.5)	<0.001 ^a^
Education level ^1^	4.12 (3.77)	4.06 (1.1)	4.2 (1.1)	0.440 ^a^
Number of children at home	1.8 (0.8)	1.8 (0.8)	1.8 (0.8)	1.000 ^a^
Income ^2^	3.3 (1.1)	3.3 (1.2)	3.2 (1.1)	0.555 ^a^
	*n (%)*			
Work status				
Not working	36 (16.4)	17 (15.6)	19 (17.3)	0.918 ^b^
Working part-time	72 (32.9)	37 (33.9)	35 (31.8)	
Working full-time	111 (50.7)	55 (50.5)	56 (50.9)	

Note. ^1^ 1 = Primary education; 2 = Lower secondary education; 3 = Higher secondary education; 4 = Bachelors; 5 Masters; 6 = Doctorate; ^2^ 1 = less than € 1500; 2 = €1500 to €2500; 3 = €2500 to €4000; 4 = €4000 to €5500; 5 = €5500 to €7000; 6 = more than €7000; ^a^ *p* value is based on a *t* test; ^b^ *p* value is based on χ^2^ test.

For replication purposes, we started by testing Hypothesis 1. Given that there was an age difference, we ran a MANCOVA with family type as the between-subjects factor and the four subscales of the parental burnout as dependent variables, controlling for the effect of age. In accordance with H1, the multivariate main effect of family type was non-significant on all the subscales of parental burnout, Pillai’s trace = 0.02, F (4, 216) = 1.19, *p* = 0.317, ηp^2^ = 0.02, observed power = 0.37. 

We then tested Hypotheses 2 and 3. In line with H2, no differences were found in balance between risks and resources as a function of family type, t (220) = 0.80, *p* = 0.425. This absence of differences cannot be attributed to a lack of statistical power. Finally, hierarchical regression models on each parental burnout subscale were run controlling for family type, age, and gender in Step 1 and balance of risks and resources in Step 2. All indicators in the regression analyses yielded results within the established cut-off values for multicollinearity (tolerance > 0.83; VIF < 1.21). In accordance with H3, the four regression models were significant and the BR^2^ was a significant predictor of all parental burnout dimensions (Table 3). 

## 4. General Discussion

This paper aimed to investigate differences in parental burnout and its antecedents among same-sex and different-sex families. The findings suggest that family type does not influence the global balance between risks and resources, nor the level of parental burnout. As previously found, the balance between risks and resources is a significant predictor of all parental burnout dimensions. 

Our findings are in line with decades of studies which have reported more similitudes than differences between same-sex and different-sex family dynamics [20,21,22,23,24,25]. Balance between risks and resources (BR^2^) was not associated with family type and was a significant predictor of parental burnout, independently of family type, gender, or age. This result is in accordance with the findings of Mikolajczak et al. [7], according to which psychological variables, rather than sociodemographic features seem to explain a greater proportion of parental burnout. However, research has suggested that same-sex families are faced with specific risks [12,13,14,15,16] and resources [30,31,32,33] and a thorough assessment of the specific risks and resources of these families (i.e., a focus on the content and not only the global balance) would be worthwhile. Future works can also move beyond a comparative framework [28,29], and instead of using a different-sex family control group, take into account specificities of same-sex parent families. Furthermore, there is growing evidence that, in some instances, same-sex families may outperform different-sex ones [27], and lower levels of parental burnout among the first would not be a surprising result in the future.

This is the first study to explore parental burnout among same-sex families. Another strength relates to the use of rigorously matched samples of participants. Still, there are some caveats that need to be taken into account, such as the low statistical power of Study 1, the leptokurtic distribution of the subscale Contrast, and the relative homogeneity of sample 2 in terms of SES. Another limitation relates to the removal of stepfamilies from the sample of different-sex families. In fact, step-parenthood is very common among same-sex families, and discarding step-families from the different-sex sample might have introduced a mismatch between the two samples. However, stepfamilies were excluded because the “coparenting” items of the BR^2^ instrument could be interpreted differently by parents in a stepfamily. Authors are in the process of developing and validating a version of BR^2^ appropriate for stepfamilies. In the meantime, it was scientifically more rigorous to remove stepfamilies from the sample to ensure that all parents interpreted BR^2^ items in the same way. Findings should thus be considered exploratory, and the robustness of the conclusions needs to be replicated among a larger and more balanced sample in terms of family configuration (e.g., stepfamilies), gender, SES, or geographic provenience.

## 5. Conclusions

Parental sexual identity is associated neither with the global balance between risks and resources nor with levels of parental burnout. In the future, attention should be given to specific risks and resources among same-sex families which might differentially affect their parental burnout levels. 

## Figures and Tables

**Table 1:** Sociodemographic Variables as a Function of Family Type (Study 1).

	*M (SD)*			
	Total (*n* = 114)	Same-Sex Families (*n =* 57*)*	Different-Sex Families *(n =* 57*)*	*p*
Age	36.9 (7.4)	37.3 (8.2)	36.5 (6.6)	0.546 ^a^
Education level ^1^	16.2 (3.8)	16.3 (3.7)	16.0 (3.9)	0.711 ^a^
Number of children at home	1.6 (0.7)	1.5 (0.7)	1.6 (0.6)	0.491 ^a^
	*n (%)*			
Neighbourhood				
Relatively disadvantaged	5 (4.4)	4 (7.0)	1 (1.8)	0.323 ^b^
Average	85 (74.6)	40 (70.2)	45 (78.9)	
Relatively prosperous	24 (21.1)	13 (22.8)	11 (19.3)	
Paid professional activity				
Yes	94 (82.5)	49 (86.0)	45 (12.0)	0.325 ^b^
No	20 (17.5)	8 (14.0)	12 (21.1)	

Note. ^1^ Years of education. ^a^ *p* value is based on a t test; ^b^ *p* value is based on χ^2^ test.

**Table 3:** Hierarchical Regression Analyses for Variables Predicting Parental Burnout Subscales (Study 2).

	Exhaustion					Contrast					Feelings of Being Fed up					Emotional Distancing				
	$R^{2}$	$\Delta R^{2}$	B	SEB	β	$R^{2}$	$\Delta R^{2}$	B	SE B	β	$R^{2}$	$\Delta R^{2}$	B	SE B	β	$R^{2}$	$\Delta R^{2}$	B	SE B	β
Step 1	0.012					0.009					0.003					0.027				
Family type			0.83	1.64	0.04			−0.37	0.66	−0.04			0.08	0.62	0.01			0.49	0.40	0.08
Age			0.07	0.11	0.05			0.08	0.04	0.14			0.03	0.04	0.07			0.05	0.03	0.14
Gender			4.37	2.48	0.12			1.42	1.00	0.10			0.78	0.93	0.06			0.15	0.61	0.02
Step 2	0.050	0.038 *				0.073	0.064 **				0.052	0.050 *				0.056	0.029 *			
BR^2^			−0.04	0.01	−0.20 **			−0.02	0.01	−0.26 ***			−0.02	0.01	−0.23 ***			−0.01	0.003	−0.17 *

Note. Family type: 0 = different-sex family; 1 = same-sex family; BR^2^ = Balance between Risks and Resources; *** *p* < 0.001; ** *p* < 0.01; * *p* < 0.05.

## Data Availability

Not applicable.
